# IMPACT OF INTERGENERATIONAL SERVICE LEARNING ON PSYCHOLOGICAL DISTRESS FOR HOMEBOUND OLDER ADULTS DURING COVID

**DOI:** 10.1093/geroni/igac059.2916

**Published:** 2022-12-20

**Authors:** Keith Chan, Christina Marsack-Topolewski, Maggie Ratnayake, Jillian Graves, Sarah LaFave, Diane Fenske

**Affiliations:** Hunter College, City University of New York, New York City, New York, United States; Eastern Michigan University, Ypsilanti, Michigan, United States; Lori’s Hands, Newark, Delaware, United States; Eastern Michigan University, Ypsilanti, Michigan, United States; Lori’s Hands, Newark, Delaware, United States; Eastern Michigan University, Ypsilanti, Michigan, United States

## Abstract

More than 2 million of older adults are homebound and 5 million need help leaving their homes. They often experience social isolation, food insecurity, and lack of connection to community resources, which for many has intensified since the pandemic. To date, home-based services for those aging in place are lacking. Using newly available data, this study examined the benefits of an intergenerational home-based service learning program in reducing psychological distress for a community-based sample of 190 homebound older adults during the COVID-19 pandemic. Multivariate regression analyses were conducted to examine the association of living in one’s own home, disability status, presence of child and spousal caregivers, and length of services from the program with psychological distress. Findings indicated that length of service with the intergenerational in-home support program was associated with lower psychological distress (β = -0.16, p < 0.05). Having a child as a caregiver was associated with lower psychological distress (β = -0.15, p < 0.05). Poor health status was associated with higher levels of psychological distress (β = 0.16, p < 0.05). Living in one’s own home, having a spouse as a caregiver, disability status, and having a long-term medical condition were not associated with psychological distress in the analysis. Results from this study suggest that intergenerational in-home support services can help reduce psychological distress for homebound older adults. Policies and practice can support a pipeline of geriatric health professionals through innovative service learning models to benefit older adults, caregivers, and students.

